# Implementation of a Phase Detection Algorithm for Dynamic Cardiac Computed Tomography Analysis Based on Time Dependent Contrast Agent Distribution

**DOI:** 10.1371/journal.pone.0116103

**Published:** 2014-12-29

**Authors:** Carsten Kendziorra, Henning Meyer, Marc Dewey

**Affiliations:** Department of Radiology, Charité - Universitätsmedizin Berlin, Berlin, Germany; Institute of Automation, Chinese Academy of Sciences, China

## Abstract

This paper presents a phase detection algorithm for four-dimensional (4D) cardiac computed tomography (CT) analysis. The algorithm detects a phase, i.e. a specific three-dimensional (3D) image out of several time-distributed 3D images, with high contrast in the left ventricle and low contrast in the right ventricle. The purpose is to use the automatically detected phase in an existing algorithm that automatically aligns the images along the heart axis. Decision making is based on the contrast agent distribution over time. It was implemented in KardioPerfusion – a software framework currently being developed for 4D CT myocardial perfusion analysis. Agreement of the phase detection algorithm with two reference readers was 97% (95% CI: 82–100%). Mean duration for detection was 0.020 s (95% CI: 0.018–0.022 s), which was 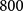 times less than the readers needed (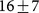
s, 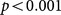). Thus, this algorithm is an accurate and fast tool that can improve work flow of clinical examinations.

## Introduction

An algorithm for detecting a specific phase, i.e. the time point of a single acquisition, within a four-dimensional (4D) cardiac computed tomographic (CT) dataset was developed. This algorithm was implemented within a software framework and its performance was evaluated.

Dynamic (4D) myocardial perfusion analysis can be performed by calculating perfusion from the time-dependent contrast agent distribution in the myocardium. KardioPerfusion (https://github.com/CardiacImagingCharite/CardiacPerfusion) is a software in development for this purpose. One early step in the analysis is the alignment of the heart. The long axis of the left ventricle (LV), which is the line through the apex of the LV and the mitral valve, will be rotated parallel to the coordinate system axis. Hence we included the AutoAlignHeart code (https://github.com/CardiacImagingCharite/AutoAlignHeart), which aligns the heart in that way. This is done by fitting the LV to an ellipsoid and considering the major ellipsoid axis as the long axis of the LV [1]. However, this algorithm requires a phase where the LV has high contrast and the right ventricle (RV) has low contrast for easier detection of the long axis of the LV. In the early stage of developing the software, the phase was manually selected by the user.

Manual phase selection requires time and concentration of the user – in most cases the radiologist performing the CT examination. Furthermore, this step needs to be performed during the examination and the physician will be disturbed. Even semi-automatic solutions, such as simple bolus tracking with a region of interest (ROI) in the LV and selecting the phase with maximum attenuation, would require user interaction at least for marking the ROI in the dataset to ensure that the ROI actually lies within the LV. Our aim was to develop a fully automatic tool not disturbing workflow by eliminating the need for physician interaction during examination. Therefore, we developed an algorithm based on the contrast agent distribution over time to automatically detect a phase with high contrast in the LV and low contrast in the RV. This algorithm is called itkFindLeftVetricle (findLV) within the software framework.

The purpose of the present study is to test if the findLV algorithm is an accurate and time-saving tool for phase detection in 4D cardiac CT analysis.

## Methods

### Algorithm Development

The main idea for automatic phase detection is to use time-dependent contrast agent distribution. The contrast agent flows from the RV through the lung to the LV. Consequently, attenuation increases over time with the phases in the LV. The algorithm takes advantage of this. It searches for the phase with the most pronounced attenuation increase compared with the first phase. Evaluation is performed in a voxelwise manner, considering only voxel positions an attenuation between 30 and 150 Hounsfield units (HU) in the first phase. This is due to the aim to consider only areas with blood and no contrast agent in the first phase. This should be particularly the case for the LV. For each considered voxel position, the maximum value of all phases is then compared to the first phase. If a phase has an attenuation increase of at least 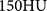, the phase number of the maximum value is stored in a one-dimensional histogram (phase histogram) with one bin for each phase. The phase with the most voxels fulfilling both conditions is chosen as the phase with high contrast in the LV and low contrast in the RV.

### Algorithm Implementation

The findLV algorithm was implemented in the KardioPerfusion software frame work. Additionally, a shrink filter was included at the beginning of the algorithm. This was done to reduce the number of voxels per image by an integer factor (shrink factor) in each dimension in order to make the algorithm faster. The shrink filter calculates the output voxel value as the mean of the corresponding input voxel values. It works multithreaded, again to speed up the algorithm. This shrink filter was originally developed by us. It was improved, thanks to the personal communication with Bradley Lowekamp, with an adaption to the itkBinShrinkImageFilter [2]. The same shrink factor was used for all dimensions. The shrink factor could be changed indirectly by the user by setting the number of voxels in x-direction for the output image. Since quadratic x, y-planes of the images, with the same number of voxels in x and y-direction, were assumed as input for the software, this quantity was called matrix size in the user interface of the software. It is also termed matrix size here.

**Figure 1:**
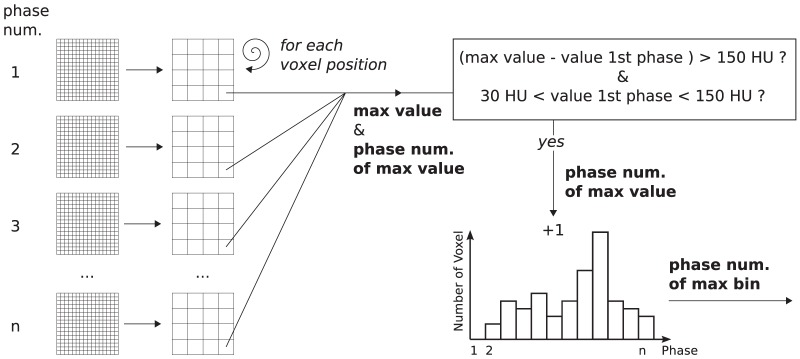
Flowchart for the findLV algorithm. First, the images are shrunk. Second, the maximum value over all phases and the corresponding phase number are evaluated for each voxel position. The maximum value has to have an increase of at least 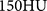 compared to the voxel in the first phase. Furthermore, the value in the first phase has to be between 30 and 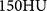 for each voxel position. If both conditions are fulfilled, the phase number is entered in the phase histogram. The phase with the maximum number of voxels in the phase histogram will be selected as the detected phase.

The algorithm includes four main steps. The first step is shrinking of the input images, and its complexity increases linearly with the number of input voxels. The second step is the loop over all voxels of the shrunken images, which is on average linear with the number of voxels of the shrunken image. For each voxel position, the maximum values over all phases and the corresponding phase number are stored in arrays during this step. The third step is analysis of these stored arrays and is done to verify if the maximum value has an increase of at least 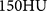 compared to the value in the first phase. Additionally, the value of the first phase must be between 30 and 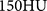. The phase number of the maximum value is entered into the phase histogram if both conditions are fulfilled. This step was linear with the number of output voxels. The last step is the single loop over the phase histogram, which is linear with the number of phases. The whole procedure is illustrated in Fig. 1.

The number of input voxels per image was fixed, since it was given by the images used for analysis. The number of phases was also fixed, since it was given by the whole dataset. Both parameters are determined by the given dataset and thus cannot be manipulated to contribute to optimization of the algorithm. Hence, the shrink factor, i.e. the matrix size of the shrunken images, was the only parameter to influence the complexity of the algorithm and in turn to optimize the performance of the algorithm.

### Data Preparation

**Figure 2:**
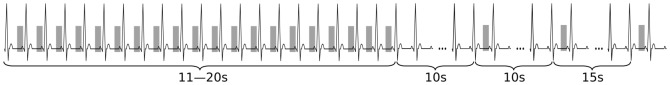
Image acquisition protocol for 4D CT myocardial perfusion analysis. Image acquisition (gray area) is done in each RR-interval during the first 11–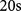. These 11–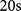 are called dynamic phase. The length depends on the heart rate with the aim to cover up to 20 RR-intervals. Three additional single acquisitions (late phases) are done 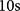, 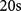, and 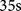 after the dynamic phase.

The study included 29 patients with a mean age of 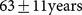 (range 42–79 years) and a clinical indication. The study was approved by the Charité Ethikkomission (local IRB), and patients gave written informed consent. CT was performed on a whole-heart coverage 320-row CT scanner (Aquilion ONE, Toshiba Medical Systems, Tochigi-ken, Japan) [3]. The contrast agent Xenetix 350 (Iobitridol 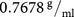) was used. 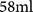 Xenetix 350 was administered at flow rate of 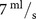, followed by 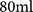 sodium chloride solution administered at the same flow rate. Images were acquired during a dynamic window, with acquisition in each RR-interval (interval between two successive maxima in the ECG wave), which was followed by three additional single acquisitions 10 s, 20 s and 35 s after this window. The specific design of the 4D CT image acquisition is illustrated in Fig. 2. Mean heart rate was 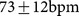 (range 49–98). The mean number of phases per patient was 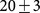 (range 14–26). Image reconstruction was performed using Adaptive Iterative Dose Reduction 3D (AIDR-3D) [4]. All images were half scan reconstructions at 75% of the RR-interval. The default value for the reconstruction field of view (FOV) of the images was 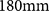. This was a standard value that ensures coverage of the whole heart in the images with highest spatial resolution [5]. For 12 patients with larger hearts, the images had larger FOVs of up to 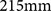. The mean value was 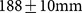. The original input images had 512×512 voxels in x-, y-direction and between 400 and 560 voxels (mean 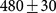) in z-direction.

To improve workflow with the use of the KardioPerfusion software, all images of each patient were shrunk with factor 4 and stored in project files. This allowed fast access to all images, although with reduced resolution, without waiting for loading from harddisk. The original images were then loaded in the background, after the project file was loaded. These pre-shrunk images with 128×128 voxels in x-, y-direction and between 100 and 140 (mean 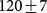) voxels in z-direction were kept in memory and considered as the input images for the findLV algorithm. This reduced the shrinking in the findLV algorithm and improved performance in terms of speed by a factor of 64.

### Test Matrix Size

The PC used to test the performance of the algorithm had an Intel Core i7-2600 3.40 GHz CPU, 16 GB RAM and ran on Linux Mint 14 64-bit operating system. The software status for this test was tagged with v0.1.2 (https://github.com/CardiacImagingCharite/CardiacPerfusion/tree/v0.1.2). For each patient, the PC ran the whole findLV algorithm with different shrink factors. The algorithm was performed with shrinking the images from 512×512 to 2×2, 4×4, 8×8, 16×16, 32×32, 64×64 and 128×128 voxels in x-, y-direction. The number of voxels in z-direction was calculated with the same shrink factor, but depended on the number of voxels in the original image. As the reference standard, two readers visually identified phases with high contrast in the left ventricle and low contrast in the RV from the 4D datasets, that should be a sufficient input for the AutoAlignHeart algorithm. This could be more than one phase per patient. The intersection of the two readers were considered as the reference phases. The criteria for the best matrix size were agreement with the readers and a short duration.

### Statistical Analysis

Data were analysed using R (http://www.r-project.org, version 3.0.1). The values for agreement and the time required for the phase detection are given as mean values with 95% confidence intervals (CI). Agreement between the detected phase provided by the algorithm with the reference phases identified by the readers results in a ratio with values of 0 to 1. Hence, the CIs were calculated with the inverse of the cumulative beta probability density function. Thus, the error bars are asymmetric. The calculation of the CIs of agreement was the only part of the statistical analysis that was done with LibreOffice Calc (http://www.libreoffice.org, version 3.6.2.2). The CIs for the time needed for phase detection were calculated assuming normal distribution. The threshold of significance was 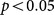. Bonferroni correction [6] was applied for evaluation of the time used for phase detection. Since there are 7 matrix sizes with 21 possible combinations, a 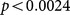 was defined as significant.

## Results

**Figure 3:**
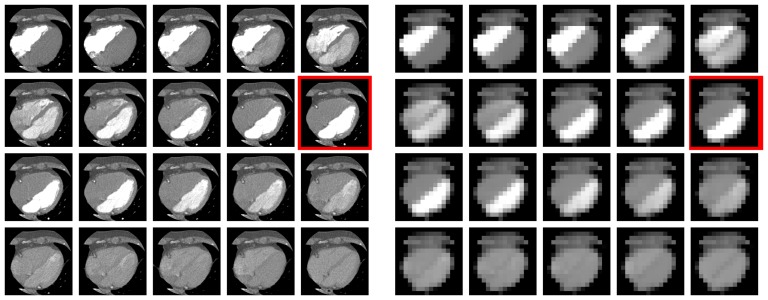
Example of an image series of one patient without shrinking (a) and with shrinking to a matrix size of 16×16 (b). All images are in axial view. The series (from top left to right) starts with already high contrast in the RV. RV contrast is low again when the LV has high contrast. The algorithm also provides a suitable phase when information in the input images is marked reduced as in (b). The 10th phase (red border) is the phase detected by the findLV algorithm.

**Figure 4:**
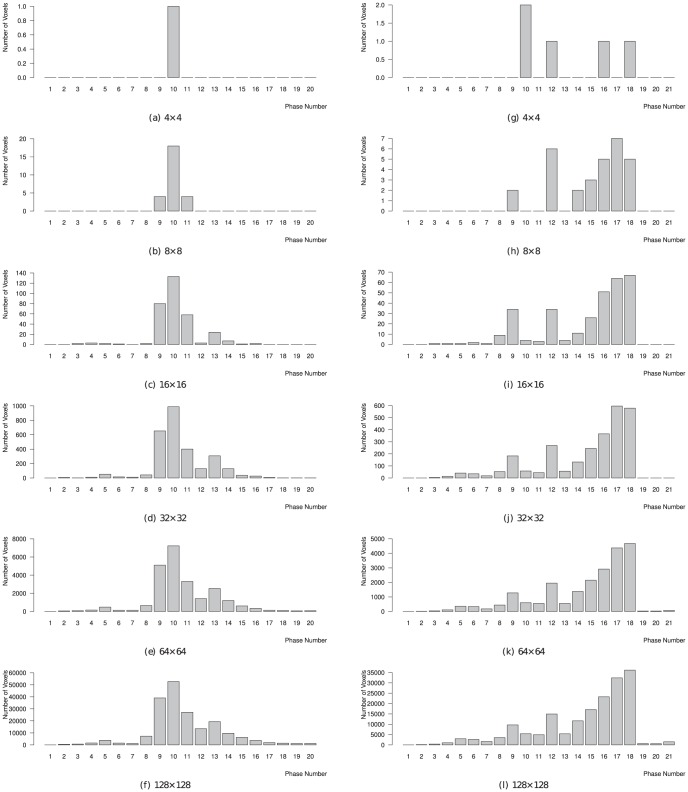
Example of phase histograms for two patients with different matrix sizes. The reference phases for the first patient (a–f) were number 9 and 10. The algorithm always detected phase number 10, in agreement with the two readers. The reference phases for the second patient (g–l) were number 16–18. The algorithm detected a wrong phase (10) for the 4×4 matrix size (g) and phase number 17 or 18 for all other matrix sizes, which agreed with the readers. The decision for the matrix size of 8×8 (h) was very tight, because the detected and matched phase number 17 counted only one voxel more than the non-agreed number 12. Accordingly, a matrix size of 16×16 is recommended.

An example of an image series and the detected phase without shrinking and with shrinking to a matrix size of 16×16 in the same patient is shown in Fig. 3. Examples of phase histograms for two patients are shown in Fig. 4.

**Figure 5:**
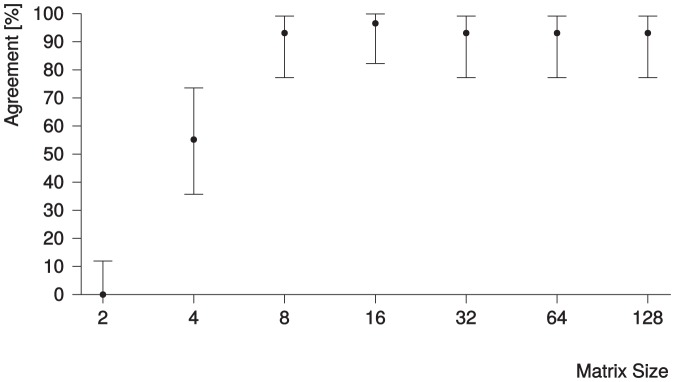
Agreement of the phase number provided by the findLV algorithm with the reference phases identified by the two readers for each matrix size investigated. The numbers on the x-axis represent the number of voxels in both the x- and y-direction of the image. The number of voxels in z-direction depends on the original image, but the same shrink factor was used for all three dimensions. 95% CIs are displayed as error bars. The readers could choose multiple phases and the intersection of both was the reference for the algorithm. The matrix size of 16×16 resulted in the highest agreement with a value of 97% (95% CI: 82–100%).

The results for agreement of the findLV algorithm with the reference readers for each matrix size investigated are shown in Fig. 5. With a matrix size of 2×2, the algorithm failed to detect a phase. Accordingly, agreement with the readers was 0% (95% CI: 0–12%). With a matrix size of 4×4, the algorithm detected a phase in 28 of 29 cases, with a agreement of 55% (95% CI: 36–77%), including all 29 cases. For matrix sizes of 8×8 and higher, the algorithm always detected a phase, and agreement with the readers was over 90% for all cases. The matrix size of 16×16 resulted in the best agreement with a value of 97% (95% CI: 82–100%), which is significantly higher than for 4×4 and 2×2 (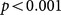) but not for the other matrix sizes (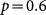).

**Figure 6:**
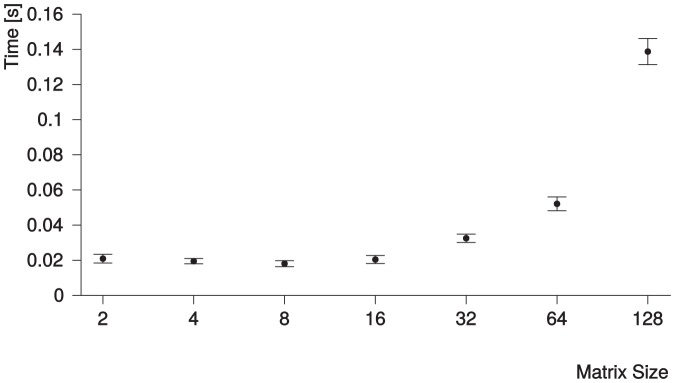
Time required by the findLV algorithm, including shrinking, for each matrix size images were shrunk to. The numbers on the x-axis represent the number of voxels in both the x- and y-direction of the image. The number of voxels in z-direction depends on the original image, but the same shrink factor was used for all three dimensions. 95% CIs are displayed as error bars. The mean time for the matrix size of 16×16 with the best agreement (cf. Fig. 5) was 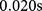 (95% CI: 0.018–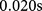).

The mean duration of phase detection per patient for different matrix sizes is shown in Fig. 6. The time needed for phase detection includes the time needed for shrinking the images from 128×128 to smaller matrices. The longest mean time was needed with a matrix size of 128×128 and was 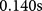 (95% CI: 0.133–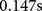). Mean time decreased for smaller matrix sizes and was lowest for a size of 8×8. This minimum mean time was 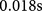 (95% CI: 0.016–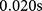). For matrix sizes of 4×4 and 2×2, the mean time was 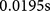 (95% CI: 0.0180–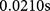) and 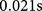 (95% CI: 0.019–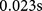), which was slightly higher compared to the minimum. The mean time for the matrix size of 16×16 with the best agreement was 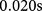 (95% CI: 0.018–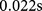). This mean time was not significantly higher than for an 8×8 matrix size (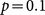). However, it was significantly lower than for a 32×32 matrix size and larger matrix sizes (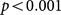).

The mean time the reference readers needed to find usable phases was 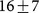
s. For all matrix sizes, the algorithm was significantly faster than the readers (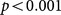). The mean time for the matrix size of 16×16 with the best agreement was 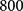 times smaller compared to the average time needed by the reference readers.

## Discussion

Our aim was to evaluate an algorithm for detecting a specific phase for 4D cardiac CT analysis. The algorithm is simple, but has good agreement with the phases manually detected by reference readers and is much faster. Furthermore, we wanted to identify an optimal matrix size of the images used by the algorithm.

Our results indicate that not only 16×16 but even a smaller matrix size of 8×8 is sufficient for the algorithm to work properly and show good agreement with the reference readers. Fig. 3 illustrates the simplicity and accuracy of the algorithm.

Matrices with a size of 4×4 voxels did not provide enough information to detect any phase in 2 of 29 cases; in another 11 cases the algorithm detected phases that did not match the reference phases selected by the readers. Matrices with 2×2 voxels did not even detect any phase. With matrix sizes of 8×8 and larger, the degree of agreement of the algorithm with the readers is mostly independent of matrix size. The information provided by these, partially very small, matrix sizes seems to be sufficient for the algorithm to work properly. Nevertheless, the decision of the algorithm was in a few cases very tight, i.e. depended on a single voxel, for 8×8 matrices (cf. Fig. 4). Although the agreement with the readers is not significantly worse for 8×8 matrices compared to 16×16 matrices, one could get the impression, that the findLV algorithm would fail more often from time to time when 8×8 instead of 16×16 would be used. Since accuracy is the most important criteria for software that is mentioned to be used in clinical routine for the future, we recommend the use of a matrix size of 16×16.

The findLV algorithm is a simple, accurate, and fast tool for the analysis of 4D cardiac CT datasets. Using a matrix size of 16×16 voxels, the algorithm showed 97% (95% CI: 82–100%) agreement with the reference readers. The corresponding mean time per patient was 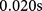 (95% CI: 0.018–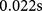) compared with 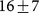
s for the reference readers. The time used by the algorithm is 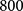 times smaller than the time needed by the readers and is thus negligible. Consequently, the time used by the readers can be saved with the use of the findLV algorithm. Furthermore, the algorithm is fully automatic and requires no user interaction. For these reasons, the findLV algorithm has potential to improve workflow in 4D cardiac CT analysis.

Cases with physiological deformations such as hearts with an eccentric LV, e.g. after myocardial infarction with aneurysm, were not included in the patient population investigated. Hence, it could not be tested if the algorithm would work properly under such conditions as well.

It was shown that the findLV algorithm provides fast evaluation of 4D datasets. Other clinical fields of application besides 4D CT myocardial perfusion analysis might also benefit from the algorithm with or without modifications. Using the 4D analysis described here (cf. Sec. Methods, Algorithm Development), the algorithm could be modified to detect other phases such as a phase with only high contrast in the RV and low contrast in the LV or with low contrast in both ventricles. Moreover, 4D magnetic resonance imaging (MRI) myocardial perfusion datasets [7] could potentially be analyzed with the algorithm. To be used for MRI, the algorithm must be adjusted to select a phase based on signal intensities rather than attenuation. Furthermore, the algorithm could be modified to detect a specific phase based on contrast enhancement in 4D CT [8]–[13] or MRI [14]–[17] acquired after contrast agent administration.

It should be noted that the algorithm was only tested with images with a FOV between 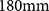 and 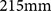. These FOVs were adapted to the size of the heart according to recommendations [5]. Images with larger FOVs, for example with a diameter of 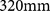 to cover the thorax, were not tested here. The heart, which is the decisive region for phase detection, would be a smaller portion in those thorax images. In turn, such images contain more but less decisive information. Furthermore, the contrast agent also passes through the lungs with enhancement varying over time. Hence, during the phase of high lung contrast, this organ might be mistaken for the LV. This is a possible limitation of the algorithm. On the other hand, larger FOVs are not suitable for cardiac CT analysis.

Another possible limitation is the acquisition time. The CT scans investigated here were acquired starting when contrast was already high in the RV (cf. Fig. 3) in order to reduce radiation exposition. Hence, the voxel positions of the RV have values larger than 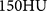 and are not considered by the algorithm (cf. Sec. Methods, Algorithm Development). If acquisition started before the contrast agent reaches the RV, those voxels would be eligible and could lead to misdetection of a phase with high contrast in the RV. A possible adaption could be to identify a phase with a first maximum and to take this phase as reference instead of the first phase. Consequently, only phases after the first maximum would then be considered for detection. Earlier acquisition would expose the patient to a higher radiation dose and should be avoided. Moreover, a benefit of this would have to be shown in further clinical studies.

The aim of the findLV algorithm is to detect a phase with high contrast in the LV and low contrast in the RV. Actually, however, the algorithm is only searching for the phase with the highest increase in attenuation. This would be the phase with the highest contrast in the LV, assuming that the acquisition starts with high contrast in the RV, as described above. The algorithm does not check, if the RV actually has low contrast. The protocol of contrast agent injection used for this clinical purpose is designed with sufficiently short injection durations (cf. Sec. Methods, Data Preparation) to ensure low contrast in the RV when the LV has high contrast (cf. Fig. 3). A contrast agent protocol with longer injection durations, as used for triple rule-out CT angiography [18], can cause high contrast in the RV when LV has its highest contrast. This could lead to misdetection of a phase with high contrast in both ventricles instead of only high contrast in the LV. However, such protocols are usually only used for triple rule-out CT angiography and are not recommended for myocardial perfusion. If such a protocol were used, the algorithm would have to be adapted in a way that it additionally checked for low contrast in the RV.

## Conclusion

A phase detection algorithm, the findLV algorithm, for 4D cardiac CT analysis was developed and implemented in the KardioPerfusion software framework. The algorithm evaluates time-dependent attenuation to detect a phase with high contrast in the LV and low contrast in the RV. It was shown that prior shrinking improves the speed of the algorithm while maintaining very good agreement with reference readers. The optimal matrix size in terms of accuracy was 16×16. The findLV algorithm is a simple, accurate and time-saving tool.
